# Supplementary material for: Genetic dissection of the Transcription Factor code controlling serial specification of muscle identities in Drosophila
Source: eLife. 2016 Jul 20;5:e14979. doi: 10.7554/eLife.14979 (PMC4954755; doi:10.7554/eLife.14979)
Supplement: Figure 2—source data 1. — DA3>DA2: complete or partial DA3 into DA2 muscle orientation; DA2>DA3: complete or partial DA2 into DA3 muscle orientation; ColexpDO5: DO5 muscle expressing Col. Loss of Col: loss of DA3 muscle Col expression. DA3>DT1 transformations have been quantified in colLCRM-moeGFP embryos. DOI: http://dx.doi.org/10.7554/eLife.14979.007 [file elife-14979-fig2-data1.docx]

|  | DA3>DA2 | DA2>DA3 | Col^exp^DO5 | Loss of Col^exp^ | Absent/misshapped DT1 | Absent /misshaped  LL1 |  | DA3>DT1 |  |
| --- | --- | --- | --- | --- | --- | --- | --- | --- | --- |
| *wt* | 0/126 | 0/126 | 0/126 | 0/126 | 0/126 | 0/126 |  | 0/52 |  |
| *aop* | 80/123 (65%) | 4/123 (3%) | 0/123 | 2/123 (<2%) | 48/123 (39%) | 47/123 (38%) |  | / |  |
| *edl* | 16/124 (13%) | 47/124 (38%) | 0/124 | 15/124 (12%) | 61/124 (49%) | 5/124 (4%) |  | / |  |
| *eya* | 82/140 (59%) | 0/140 | 0/140 | 101/140 (72%) | 8/140 (6%) | 129/140 (92%) |  | / |  |
| *noc* | 0/126 | 0/126 | 0/126 | 116/126 (92%) | 0/126 | 32/126 (25%) |  | 19/73 (26%) |  |
| *so* | 37/116 (32%) | 0/116 | 78/116 (67%) | 0/116 | 0/116 | 0/116 |  | / |  |

**Table 1. Quantification of muscle phenotypes observed in *aop*, *edl*, *eya*, *noc* and *so* mutant stage 15 embryos.**

DA3>DA2: complete or partial DA3 into DA2 muscle orientation; DA2>DA3: complete or partial DA2 into DA3 muscle orientation; Col^exp^DO5: DO5 muscle expressing Col. Loss of Col^exp^ : loss of DA3 muscle Col expression. DA3>DT1 transformations have been quantified in *col^LCRM^-moeGFP* embryos.
